# Allegiance Bias and Treatment Quality as Moderators of the Effectiveness of Humanistic Psychotherapy: Protocol for a Systematic Review and Meta-Analysis

**DOI:** 10.2196/15140

**Published:** 2019-11-25

**Authors:** Olivia Schünemann, Alessa Jansen, Ulrike Willutzki, Nina Heinrichs

**Affiliations:** 1 University of Bremen Bremen Germany; 2 Bundespsychotherapeutenkammer Germany Berlin Germany; 3 Witten/Herdecke University Witten Germany

**Keywords:** bona fide, systematic review, meta-analysis

## Abstract

**Background:**

In many countries, humanistic psychotherapy (HPT) is viewed as a broad psychotherapeutic approach and is accepted in health care systems. To qualify for reimbursement by health insurance in Germany, psychotherapy approaches have to be evaluated positively by the German Scientific Board of Psychotherapy (GSBP). The GSBP examined HPT and its subapproaches based on an application by a number of professional organizations affiliated with HPT (Work Group Humanistic Psychotherapy, WGHPT). The GSBP came to the decision that none of the HPT subapproaches provided sufficient evidence to be evaluated as evidence based. Potential reasons for the discrepancy between international recognition of HPT and GSBP’s decision will be explored: researchers’ allegiance may have led to a risk of bias disadvantaging HPT. Furthermore, the evaluation criteria of the GSBP did not systematically consider whether HPT was conceptualized bona fide and implemented with sufficient treatment integrity in the studies.

**Objective:**

This systematic review will re-examine the studies included in the review of the GSBP. Within 2 comparisons (HPT vs control and HPT vs other psychotherapeutic interventions), we will examine moderating effects of treatment quality (bona fide and treatment integrity) and allegiance on the effectiveness of HPT.

**Methods:**

This review is based on the prior systematic review by the GSBP. The GSBP examined randomized controlled trials (RCTs) and studies with non-RCTs of HPT interventions for individuals with mental disorders. All studies suggested by the WGHPT were included; moreover, the GSBP conducted searches in standard electronic databases (Cochrane Central Register of Controlled Trials, MEDLINE, PsycINFO, and PSYNDEX) and handsearches in relevant systematic reviews and contacted experts. A total of 2 independent GSBP reviewers performed study screening using a structured form. On the basis of the prior work of the GSBP, all studies that were positively screened by the GSBP will be included in this review. Data will be extracted independently by 4 authors. Standardized mean difference will be calculated, and possible publication bias will be tested using funnel plots and Egger test. A priori defined subgroup or meta-regression analyses will be performed for treatment quality, allegiance, type of nonactive control, study quality, type of subapproach, and target population (children and adolescents or adults).

**Results:**

The GSBP identified 115 eligible studies that will be reanalyzed in this systematic review.

**Conclusions:**

Results about moderator effects of treatment quality and allegiance will provide important information about their impact on the evaluation of HPT and other psychotherapy approaches and can be used for further evaluation methods.

**Trial Registration:**

PROSPERO CRD42019128983; https://www.crd.york.ac.uk/prospero/display_record.php?RecordID=128983

**International Registered Report Identifier (IRRID):**

PRR1-10.2196/15140

## Introduction

### Background

Humanistic psychotherapy (HPT) is often viewed as a broad psychotherapeutic approach next to psychodynamic, cognitive behavioral, and systematic psychotherapeutic approaches [1], and HPT is largely accepted worldwide. However, in Germany, as yet, HPT is not approved within the German health care system—in contrast to psychodynamic, cognitive behavioral, and systemic therapy. To become established in the German health care system and for service providers (eg, psychotherapists) to be reimbursed by health insurances, the procedure is as follows: the German Scientific Board of Psychotherapy (GSBP) in a first step evaluates the concept and evidence of psychotherapeutic approaches or subapproach methods. Only if the GSBP recognizes an approach as scientifically sound, further steps to integrate the approach into the German health care system are taken. In 2012, a number of professional organizations affiliated with HPT, the Work Group Humanistic Psychotherapy (WGHPT), submitted an application for scientific recognition of HPT (Humanistic Psychotherapy Application, HPT-A) to the GSBP [2]. On the basis of the GSBP’s method paper 2.8 [3] that defines criteria and procedures for the evaluation process, the GSBP examined HPT, including its subapproaches as defined by the HPT-A [2].

The general evaluation process within the GSBP includes 2 steps. First*,* it is evaluated whether the concept of an intervention can be judged as a psychotherapy approach (an overall concept) or as a subapproach according to the criteria of the GSBP. Second, the evidence for the approach or its subapproaches is evaluated. The GSBP concluded in 2018 that HPT may not be considered as a psychotherapy approach according to its criteria [4]. This was mainly decided because of the heterogeneity of the HPT subapproaches and the lack of a common concept of indications and contraindications; furthermore, a general training concept for HPT as a broad concept including knowledge about all subapproaches was not sufficiently elaborated. As a consequence, GSBP evaluated the evidence on the level of HPT subapproaches but not as an overall approach [4]. None of these subapproaches provided enough evidence to be evaluated as evidence based according to the criteria of the GSBP [4]. Moreover, even when considering all subapproaches together, there was not sufficient evidence for the treatment of anxiety disorders with HPT; this is a mandatory requirement for the recognition of a psychotherapy approach by the GSBP [4].

This conclusion was much debated within the German professional public. In light of the widespread recognition of HPT worldwide, this outcome seems puzzling: other reviews on HPT [5] and the meta-analysis by Elliott et al [1] mainly demonstrated positive effects of HPT. Elliott et al [1] analyzed about 191 studies on the effectiveness (including both efficacy and effectiveness studies) of HPT involving person-centered psychotherapy, supportive or nondirective psychotherapy, task-focused psychotherapy, integrative emotion-focused psychotherapy, existentially oriented supportive-expressive group therapy for medical populations (eg, cancer), and other subapproaches (such as gestalt psychotherapy or psychodrama) with results demonstrating large pre-post and pre–follow-up effect sizes. Results comparing only HPT with cognitive behavioral therapy (CBT) indicated that CBT seems to be slightly superior to HPT [1].

Reasons for the discrepancy between the meta-analysis by Elliott et al [1] and the result of the review process by the GSBP [4] are multifold. A central point is the nonidentical selection of subapproaches included by the WGHPT [2] versus 2 meta-analyses by Elliot et al [1,6]. Moreover, although there is some overlap between the diagnostic groups examined by Elliott et al [1] and defined by the GSBP [3], the GSBP follows the diagnostic system, for example, of the International Statistical Classification of Diseases, Tenth Revision (ICD-10), more stringently. Furthermore, the meta-analysis by Elliott et al [1] included studies applying motivational interviewing but did not examine child and adolescent psychotherapy in contrast to the GSBP [4].

Furthermore, according to the GSBP’s method paper 2.8 [3], the body of evidence is evaluated in a 2-step process: (1) does the particular study fulfill specified inclusion criteria? (see section *Criteria for Selecting Studies for This Review*) and (2) does the study provide evidence for the efficacy of the approach or subapproach: yes or no? In contrast to this categorical approach, Elliott et al [1] also accounted for the size of effects by summarizing effect sizes across studies.

When evaluating the evidence of HPT according to the GSBP procedures [4], the humanistic subapproaches were often designed as a control group in comparison with other psychotherapeutic approaches, such as CBT, in the studies that were included. As researchers’ allegiance is often associated with therapy outcomes [7], this design may have led to a risk of bias diminishing possible effects of HPT. Elliott et al [1] controlled allegiance in their comparison of HPT with CBT. This led to a reduction of the difference in pre-post effect sizes between HPT and CBT, resulting in similar pre-post effect sizes for both CBT and HPT.

In addition, the evaluation process of the GSBP [4] does not systematically evaluate the effects of treatment quality, including bona fide psychotherapy and treatment integrity. Bona fide psychotherapy may be defined by mentioning or describing an established psychological approach, psychological treatment principles, a treatment manual, or active treatment ingredients [8]. Moreover, bona fide psychotherapy is usually implemented by a trained therapist (in this case in HPT) [9]. Bona fide thus targets the *conceptual quality* of a treatment. An additional quality feature of psychotherapy is treatment integrity “[…] conceptualized broadly including adherence to specific treatment procedures (e.g., the importance of exposure in psychotherapy for post-traumatic stress disorder), common factors (e.g., therapeutic alliance), and therapist effects (i.e., differences in the effects due to individual therapists)” (pg 8 [10]). Treatment integrity thus targets the *process quality* of treatment and psychotherapy. We assume that process quality and conceptual quality are closely associated. Treatment quality seems to be relevant for research on HPT, as Elliott et al [1] found that only nondirective supportive (non–bona fide) HPT showed worse outcomes in comparison with CBT.

Thus, both allegiance and treatment quality may have had effects on the evaluation of the evidence of the humanistic subapproaches by the GSBP.

### Objectives

This systematic review will re-examine the studies identified within the systematic review of the GSBP [4] to analyze the relevance of allegiance and treatment quality (bona fide psychotherapy and treatment integrity) for the effectiveness of HPT (including both efficacy and effectiveness studies). Therefore, 2 comparisons will be conducted: (1) comparison of humanistic subapproaches versus control and (2) comparison of humanistic subapproaches versus other psychotherapeutic interventions. Within both comparisons, we will analyze the moderating effects of treatment quality. In addition, allegiance to HPT in the first comparison and allegiance to the comparator psychotherapeutic intervention in the second comparison will be analyzed as moderators.

## Methods

### Design and Procedure

This study is a secondary moderator analysis based on a systematic review conducted by the GSBP [4]. The following tasks were completed by the GSBP [4]: (1) development of a search strategy with defining criteria for the study selection (see section *Criteria for Selecting Studies for This Review*) including the types of studies, participants, interventions, and comparators (see corresponding paragraphs below); (2) search of studies (see sections *Search Methods for Identification of Studies* and *Bibliographic Database Search*); and (3) study screening (see section *Criteria for Selecting Studies for This Review*). Thus, the criteria for selecting studies and the search methods for identifying studies were defined by the methods underlying the evaluation process of the GSBP [3]. Randomized controlled trials (RCTs) and non-RCTs with control groups were included.

On the basis of the prior work of the GSBP [4], this study will pursue the following tasks: (1) data extraction according to a coding protocol developed specifically for this study (see sections *Types of Outcome Measures* and *Data Extraction*), (2) assessment of methodological quality, and (3) data synthesis (see the corresponding paragraphs below) to estimate effect sizes and potential moderators (allegiance and treatment quality).

#### Criteria for Selecting Studies for This Review

This systematic review will be based exactly on the set of studies previously identified by the GSBP. The research questions of this study focus on determining whether the evaluation of psychotherapy studies by the GSBP is biased by allegiance or treatment quality. As the board’s decisions have far-reaching consequences for psychotherapeutic approaches and their implementation in the German health care system, it is an extremely important question to evaluate whether researcher allegiance and treatment quality are to be considered or can be neglected in this context. To be able to compare the present result with the original result of the GSBP and to evaluate whether the GSBP review should be supplemented by an analysis of effect sizes and potential moderators (allegiance and treatment quality), the body of studies to be included has to be identical. There will be no additional study search to identify more recently published literature. The criteria for study selection and the search strategy of the GSBP [4] were as follows: the GSBP included and excluded studies based on a 2-step screening process. First, titles and abstracts were screened independently by 2 reviewers. Second, potentially relevant German or English publications were screened by 2 independent reviewers using a structured form [3]. Disagreement was resolved by a consensus discussion by members of the GSBP.

#### Types of Studies

The GSBP [4] examined RCTs and non-RCTs. Studies without any control group were excluded. All studies had to include pre- and postassessments regardless of follow-up assessments. Studies were excluded in case of clear indication of data manipulation.

#### Types of Participants

Studies examining participants without any mental disorder or studies assessing mental disorders without objective and reliable diagnosis process via standard operationalized diagnostic interviews were excluded. Only studies applying interventions to individuals with clinically significant mental disorders were analyzed. All diagnoses had to be made on the basis of either the ICD [11] or the Diagnostic and Statistical Manual of Mental Disorders [12]. Participants were adults or children and adolescents suffering from any of the following mental disorders (diagnostic groups according to GSBP [3]): (1) mood and affective disorders (F3, F94.1, and F53 according to ICD-10); (2) anxiety disorders and obsessive-compulsive disorders (F40-F42, F93, and F94.0); (3) dissociative and conversion disorders (F44-F48); (4) substance abuse and dependence (F1 and F55); (5) personality and behavior disorders (F6); (6) reaction to severe stress and adjustment disorders (F43); (7) eating disorders (F50); (8) nonorganic sleep disorders (F51); (9) sexual dysfunction (F52); (10) psychological and behavioral factors associated with disorders or diseases classified elsewhere (F54); (11) schizophrenia, schizotypal, and delusional disorders (F2); (12) organic, including symptomatic, mental disorders (F0); (13) mental retardation (F7) and pervasive developmental disorders (F84); (14) hyperkinetic disorders (F90) and conduct disorders (F91 and F94.2-F94.9); (15) disorders of psychological development (F80-F83); (16) nonorganic enuresis (F98.0) and nonorganic encopresis (F98.1); (17) feeding disorder of infancy and childhood (F98.2); and (18) tic disorders (F95) and stereotyped movement disorders (F98.4).

#### Types of Interventions

The following HPT subapproaches were examined according to the definitions by the WGHPT [2]: (1) client or person-centered therapy, (2) gestalt therapy, (3) emotion-focused individual and couple therapy, (4) psychodrama, (5) logotherapy, (6) existential analysis, (7) body psychotherapy (including bioenergetic, biodynamic, biosynthesis psychotherapy, and Hakomi; excluding Reiki, Alexander Technique, Feldenkrais, and breathing therapy), (8) Pesso Boyden System Psychomotor, (9) integrative therapy, and (10) transactional analysis. A manual, a treatment guideline, or the name of the psychotherapeutic subapproach had to be mentioned for the study to be included.

#### Types of Comparators

Both controlled and comparative effectiveness studies were included. Active control groups were other evidence-based psychotherapeutic interventions previously considered to be effective by the GSBP (CBT, psychodynamic psychotherapy, and systemic therapy). Nonactive comparators were no-treatment control (patients are administered only assessments); wait-list control (patients received treatment following the study period); attention-placebo, nonspecific control, sham treatment (patients received treatment that involves nonspecific psychotherapeutic factors), and treatment as usual. In addition to the GSBP [4], this review will extract *other* active and nonactive control conditions (eg, medication).

#### Types of Outcome Measures

The primary efficacy outcome of this review for the planned moderator analyses will be symptom severity at the end of treatment measured on a metric symptom-specific scale. If studies report more than 1 symptom-specific metric outcome, outcomes on self-rating scales (eg, Beck Depression Inventory) will be given priority over observer-rated scales (eg, Hamilton Depression Rating Scale). As studies on multiple populations quite likely using a broad variety of symptom-specific scales are included, it is not possible to specify a list of relevant outcomes or define a hierarchy of symptom scales before the extraction process. Therefore, first, all outcome measures from all studies will be extracted. In a second step, a sequence for all used self-rating (as well as observer-rated) scales will be determined that will be applied to all studies. In this sequence, more commonly used symptom scales and more reliable scales will be given priority over seldom used respectively less reliable scales. The sequence of scales will be documented to rule out selective outcome reporting.

Secondary efficacy outcomes will include assessment of impairment and consequences, such as interpersonal outcomes (eg, Dyadic Adjustment Scale), general assessment of functioning (eg, General Assessment of Functioning), or quality of life (eg, WHO Quality of Life).

The primary efficacy outcome will be analyzed separately for short term (end of intervention) and follow-up if available (6 months after the end of intervention). If multiple follow-up measures are reported, the one closest to 6 months will be used.

The primary safety outcome will be dropping out of the study because of any reason.

All outcomes that are likely to be meaningful to people making a decision about the target condition (clinicians, patients or consumers, the general public, administrators, and policy makers) will be addressed independently of the frequency of their reporting in primary studies. Owing to the long tradition of psychotherapy research, most instruments used in clinical trials are usually psychometrically sound. Such measures will be preferred throughout the review (if referenced or sufficient psychometric quality reported).

In accordance with the GSBP’s method paper 2.8 [3], studies that do not use any reliable or valid outcome measure (at least for primary outcomes) or that use only primary outcomes not representing relevant variables for participants or mental disorders (eg, severity of symptoms, psychological strain, impairment in daily life, quality of life, and utilization of health services) will be excluded. In addition, studies with incomplete reporting of intervention effects or changes in primary and secondary outcomes compared with the control group will be excluded.

### Search Methods for Identification of Studies

This systematic review will be based on all studies identified within the systematic review of the GSBP [4]. First, the GSBP included all studies suggested by the WGHPT [2]. Second, the GSBP used several methods to retrieve further potentially relevant articles. In addition to standard electronic databases, handsearch in relevant systematic reviews was performed. In addition, experts were contacted once in the beginning of the process and after each electronic database research to name relevant studies to add relevant missing studies.

#### Bibliographic Database Search

The following databases were searched: Cochrane Central Register of Controlled Trials, MEDLINE, PsycINFO, and PSYNDEX. No language restrictions were applied. All databases were searched using both standard vocabulary (eg, Medical Subject Headings) and keywords (freetext). The full search strategy is presented in Multimedia Appendix 1. Both English and German keywords were used. First Bibliographic database search was conducted in December 2013, and a search update was performed in July 2016. As described above, a further update of the GSBP search is not feasible as answering the primary research questions necessitates exactly the identical study pool of the GSBP [4].

### Data Collection and Assessment of Methodological Quality

#### Data Extraction

For each study, study characteristics and results for the planned moderator analyses will be extracted by 1 of 2 authors (OS or AJ) using a structured form and assessing the full text. Methodological quality for each study will be assessed independently by 2 authors (OS and NH or AJ and UW). Disagreement will be recorded and resolved by discussion either within the author pair or within whole author team if necessary. Extracted data will include information on participant characteristics (eg, age, gender, and diagnostic group), study characteristics (eg, sample sizes, study design, allocation, and dropout rates), intervention characteristics (eg, HPT subapproach, type of control group, therapist characteristics, and setting), primary and secondary outcomes, risk of bias, allegiance, and treatment quality (bona fide and treatment integrity). Outcomes will be extracted from publications with estimation and substitution of missing data according to the guidelines of the Cochrane Collaboration [13], for example, calculating standard errors from exactly reported *t* test values. Data will be managed using Microsoft Excel (Microsoft Corporation).

#### Assessment of Methodological Quality

Internal validity will be assessed with the second version of Cochrane’s risk of bias tool (RoB 2.0; [14]) adapted according to suggestions made by Munder and Barth [10] for its use in psychotherapy outcome research. The risk of bias tool provided by Cochrane is a widely used measure to assess internal validity in controlled trials, yet its application on psychotherapeutic studies has been criticized [10]. Munder and Barth [10] have therefore provided suggestions for its use in the context of psychotherapy research. Accordingly, the following 4 domains will be assessed in accordance with RoB 2.0: (1) bias arising from the randomization process (sequence generation, allocation concealment, and baseline differences), (2) bias because of missing outcome data (availability of outcome data), (3) bias in outcome measurement (appropriate measuring and differences between groups), and (4) bias in selection of the reported result (accordance with specified plan, multiple measurement, and multiple analyses). The domain “bias due to deviations from intended interventions” (pg 17 [14]; effect of adhering to intervention) of the RoB 2.0 tool will be adapted as suggested by Munder and Barth [10] (concomitant treatments, implementation of treatment, and adherence to intervention). In case of non-RCTs, risk of bias will be assessed with the ROBIN-I tool provided by Cochrane for non-RCTs comparing different interventions [15]. Allegiance will be assessed according to the multilevel allegiance rating scale provided by Steinert et al [16]. Both bona fide psychotherapy and treatment integrity will be rated using the definition by Benish et al [8] and Munder and Barth [10].

External validity (generalizability) will be addressed by documenting study setting, patient selection criteria, patient characteristics, applicability of the intervention in routine care, clinical relevance of outcomes, efficacy at follow-up, and discontinuation rates.

If considerable methodological heterogeneity is present, subgroup analyses will be performed by comparing the findings between studies of low, some, and high risk of bias (according to Munder and Barth [10]). The strength of the body of evidence will be provided by presenting and discussing results of the methodological quality of all included studies.

### Data Synthesis

#### Planned Treatment Comparisons

Overall, 2 comparisons will be conducted. First, HPT subapproaches will be compared with nonactive controls. Second, HPT subapproaches will be compared with other psychotherapeutic interventions that have previously been scientifically recognized by the GSBP (CBT, psychodynamic psychotherapy, and systemic psychotherapy).

#### Meta-Analysis

The statistical analysis will follow actual guidelines [13,17,18]. For metric measures (eg, symptom severity and quality of life), standardized mean difference will be calculated, as it is unlikely that all studies administer the same measures. For rare outcomes (dropout rates) with highly varying baseline rates, odds ratios will be calculated. For all studies, effect sizes will be calculated using the intention-to-treat principle, that is, analyzing all subjects allocated to a study arm. For all metric outcomes, the definition of the intention-to-treat sample provided by the authors will be followed if available. All analyses will be performed by applying a random effects model with inverse variance weights [19]. We plan to use a random effects model rather than fixed effects one because we assume that the included studies will not be functionally equivalent and will show considerable clinical (concerning population and intervention) and methodological (eg, design and quality) heterogeneity. Statistical heterogeneity between study results will be tested for significance using Cochran Q test and quantified using the *I*^2^ statistic [20]. Results will be visually displayed as forest plots. Possible publication bias will be tested using visual examination of funnel plots and applying Egger test [21].

#### Subgroup and Meta-Regression Analysis

A priori defined subgroup (in case of categorical predictors) or meta-regression (in case of metric predictors) analyses will be performed according to treatment quality, allegiance, type of nonactive control (wait-list vs all others including treatment as usual), study quality, type of HPT subapproach, and population (children and adolescents vs adults). Differences between subgroups will be tested formally [22-24]. All meta-regression analyses will be performed using the restricted maximum likelihood estimate method, a recommended random effect approach that accounts for residual between-trial heterogeneity [25]. In case of considerable heterogeneity between study results that cannot be explained by the a priori defined subgroup and meta-regression analyses, a series of a posteriori (explorative) meta-regression analyses will be performed to identify sources of heterogeneity. A priori and a posteriori analyses will be clearly labeled as such.

#### Sensitivity Analysis

Sensitivity analyses will be performed for the primary efficacy outcome using results from all trials in contrast to results from RCTs only.

#### Qualitative Summary

If clinical or methodological heterogeneity of the included studies proves to be extremely high, a qualitative rather than quantitative synthesis of the evidence will be performed.

## Results

This systematic review and meta-analysis was submitted for registration in the PROSPERO International prospective register of systematic reviews (CRD42019128983). As this review and meta-analysis is a reexamination of the previous review process by the GSBP, preliminary searches, piloting of the study selection process, and formal screening of search results against eligibility criteria were completed. In the context of their screening process, the GSBP identified 115 eligible studies that will be reanalyzed in the following systematic review and meta-analysis. The next step will be final data extraction.

## Discussion

The examination of the moderating effects of treatment quality and allegiance will provide important information concerning their effects on the evaluation of psychotherapy approaches. This information is crucial for the further development of the evaluation methods of the GSBP and for other stakeholders that need to assess the efficacy and effectiveness of psychotherapeutic approaches within health care systems. The examination of treatment quality and allegiance within the evaluation of humanistic subapproaches may further highlight the role of implementation of interventions when new innovative concepts are compared with well-established interventions.

## Appendix

Multimedia Appendix 1 Full search strategy.

## Abbreviations

CBT: cognitive behavioral therapy
GSBP: German Scientific Board of Psychotherapy
HPT: humanistic psychotherapy
HPT-A: Humanistic Psychotherapy Application
ICD-10: International Statistical Classification of Diseases, Tenth Revision
RCT: randomized controlled trial
WGHPT: Work Group Humanistic Psychotherapy
