# Supplementary material for: Allegiance Bias and Treatment Quality as Moderators of the Effectiveness of Humanistic Psychotherapy: Protocol for a Systematic Review and Meta-Analysis
Source: JMIR Res Protoc. 2019 Nov 25;8(11):e15140. doi: 10.2196/15140 (PMC6902128; doi:10.2196/15140)
Supplement: Multimedia Appendix 1 [file resprot_v8i11e15140_app1.pdf]

Allegiance bias and treatment integrity as moderators of the effectiveness of humanistic psychotherapy: A systematic review and meta-analysis protocol

Dr. Olivia Schünemann[1], Dr. Alessa Jansen[2], Prof. Dr. Ulrike Willkutzki[3], Prof. Dr. Nina Heinrichs[1]

[1]University of Bremen, Germany

[2]Bundespsychotherapeutenkammer, Germany

[3]Witten/Herdecke University, Germany

Supplementary material

Supplementary material

1. Full search strategy (by the German Scientific Board of Psychotherapy; GSBP)

Full search strategy

beziehungsorient* AND Psychotherap*

bioenerg* AND (therap* OR psychotherap*)

biodynam* AND (therap* OR psychotherap*)

body AND psychotherap*

body AND therap*

clarification-orient* AND psychotherap*

client-cent* AND (therap* OR psychotherap*)

drama AND therap*

Dialogische Therapie

emotional AND (role-playing OR (role AND playing))

emotional AND focused AND therap*

emotional disclosure therapy

emotion focused therapy

emotion-focused AND experiential therapy

emotionsfokussierte AND (Therap* OR Psychoth*)

erfahrungsorient* AND (Therap* OR Psychoth*)

erlebnisaktivierend* AND (Therap* OR Psychoth*)

experiencing

experiential AND (therap* OR intervention)

existential AND analys*

Existenzanalys*

experienzielle AND (Psychotherap* OR Therap*)

Fokusing OR focusing

Gestalttherap* OR (gestalt AND therap*)

gestalt group therapy

gestalt play therapy

Gesprächspsychoth* OR Gesprächsthera*

humanistic-experiental AND therap*

humanistische AND therap*

humanistic AND therap*

Kinderspieltherap*

Klientzent* OR klientzentr* AND (Psychoth* OR Therap* OR Gruppenthera*)

Klärungsorientierte AND (Gesprächstherap* OR Therap*)

Logotherap* OR Logo therap*

nondirektive OR non-directive OR nondirective AND Therap* nonprescriptive AND (treatment OR therap*)

paradoxical AND intention

paradoxe AND intervention

patient-cent*

(personenzentrierte OR personzentr*) AND (Therap* OR Psychoth*)

(person centred OR person centered) AND therap*

play AND therap*

Prozess-Erlebnisorientierte AND Psychotherap*

prozessorientierte AND therap*

process experiential AND (therapy OR intervention OR psychotherapy)

Psychodrama OR Psychodramath* OR (psychodrama AND therapy) OR (psychodramatic AND psychoth*)

Spieltherap* OR Therapeut*) AND Spiel Transaktionsana* OR transactional AND analysis
